# Multidimensional characteristics, prognostic role, and preoperative prediction of peritoneal sarcomatosis in retroperitoneal sarcoma

**DOI:** 10.3389/fonc.2022.950418

**Published:** 2022-10-27

**Authors:** Yang Li, Jian-Hui Wu, Cheng-Peng Li, Bo-Nan Liu, Xiu-Yun Tian, Hui Qiu, Chun-Yi Hao, Ang Lv

**Affiliations:** Key Laboratory of Carcinogenesis and Translational Research (Ministry of Education/Beijing), Department of Hepato-Pancreato-Biliary Surgery/Sarcoma Center, Peking University Cancer Hospital & Institute, Beijing, China

**Keywords:** peritoneal sarcomatosis, retroperitoneal sarcoma, characteristics, prognostic role, risk factors, preoperative prediction, nomogram

## Abstract

**Background:**

Peritoneal sarcomatosis (PS) could occur in patients with retroperitoneal sarcomas (RPS). This study aimed to expand the understanding of PS on its characteristics and prognostic role, and develop a nomogram to predict its occurrence preoperatively.

**Methods:**

Data of 211 consecutive patients with RPS who underwent surgical treatment between 2011 and 2019 was retrospectively reviewed. First, the clinicopathological characteristics of PS were summarized and analyzed. Second, the disease-specific survival (DSS) and recurrence-free survival (RFS) of patients were analyzed to evaluate the prognostic role of PS. Third, preoperative imaging, nearly the only way to detect PS preoperatively, was combined with other screened risk factors to develop a nomogram. The performance of the nomogram was assessed.

**Results:**

Among the 211 patients, 49 (23.2%) patients had PS with an incidence of 13.0% in the primary patients and 35.4% in the recurrent patients. The highest incidence of PS occurred in dedifferentiated liposarcoma (25.3%) and undifferentiated pleomorphic sarcoma (25.0%). The diagnostic sensitivity of the preoperative imaging was 71.4% and its specificity was 92.6%. The maximum standardized uptake value (SUVmax) was elevated in patients with PS (P<0.001). IHC staining for liposarcoma revealed that the expression of VEGFR-2 was significantly higher in the PS group than that in the non-PS group (P = 0.008). Survival analysis (n =196) showed significantly worse DSS in the PS group than in non-PS group (median: 16.0 months vs. not reached, P < 0.001). In addition, PS was proven as one of the most significant prognostic predictors of both DSS and RFS by random survival forest algorithm. A nomogram to predict PS status was developed based on preoperative imaging combined with four risk factors including the presentation status (primary vs. recurrent), ascites, SUVmax, and tumor size. The nomogram significantly improved the diagnostic sensitivity compared to preoperative imaging alone (44/49, 89.8% vs. 35/49, 71.4%). The C-statistics of the nomogram was 0.932, and similar C-statistics (0.886) was achieved at internal cross-validation.

**Conclusion:**

PS is a significant prognostic indicator for RPS, and it occurs more often in recurrent RPS and in RPS with higher malignant tendency. The proposed nomogram is effective to predict PS preoperatively.

## Introduction

Retroperitoneal sarcomas (RPSs) are rare tumors accounting for approximately 0.15% of malignancies and 15% of soft tissue sarcomas (1). They are complex family of tumors comprising of ver 60 histological subtypes, representing the full spectrum of malignant behavior (2). Peritoneal sarcomatosis (PS) is a state of intraperitoneal dissemination of sarcomas. The presence of pathologically confirmed lesions on the surface of the peritoneum or intraperitoneal viscera is considered as PS. Different from multifocal disease, which is defined as the presence of more than one noncontiguous tumor (3), PS is featured by the intraperitoneal noncontiguous tumors not covered by peritoneum or other organs. On the other hand, if the RPS has a component that penetrates the peritoneum which is contiguous with the main tumor mass, it could be defined as intraperitoneal invasion of intraperitoneal component, which is also not exactly the same as PS (4).

PS occurs only in approximately 10% of patients with primary RPS disease (5). However, it is common in patients with recurrent RPS disease, occurring in 35%–82% of patients (6, 7). It can be a spontaneous phenomenon or caused by iatrogenic factors (8). Due to the overall limited effect of pharmacotherapy on most of subtypes, surgery remains the mainstay of management for RPS (9). However, according to the consensus of the Trans-Atlantic Retroperitoneal Sarcoma Working Group (TARPSWG), the surgical indication for PS is limited to palliative intervention as dictated by symptoms (10). The role of surgery on PS remains controversial, and it is still a challenge of identifying the presence of PS prior to surgery (11). Imaging evaluation is nearly the only way to detect PS preoperatviely, but its diagnostic sensitivity is unsatisfactory. Some studies showed that the prognosis of patients with PS remains dismal, with a median survival of approximately 1 year (12, 13). Therefore, the management of PS is confronted with huge challenge and deserves more attention.

High-quality research on PS caused by RPS is limited owing to its rarity and complexity, and the understanding of its characteristics and prognostic role remains inadequate. Therefore, this study aimed to summarize the clinicopathological characteristics of PS, explore its influence on prognosis, and develop an effective preoperative nomogram to predict the occurrence of PS.

## Materials and methods

### Patients and data collection

This study was approved by the Ethics Committee of Peking University Cancer Hospital and performed according to the 1975 Helsinki Declaration and its later amendments or comparable ethical standards. All patients provided written informed consent before surgery for the use of their anonymized data.

**Figure 1:**
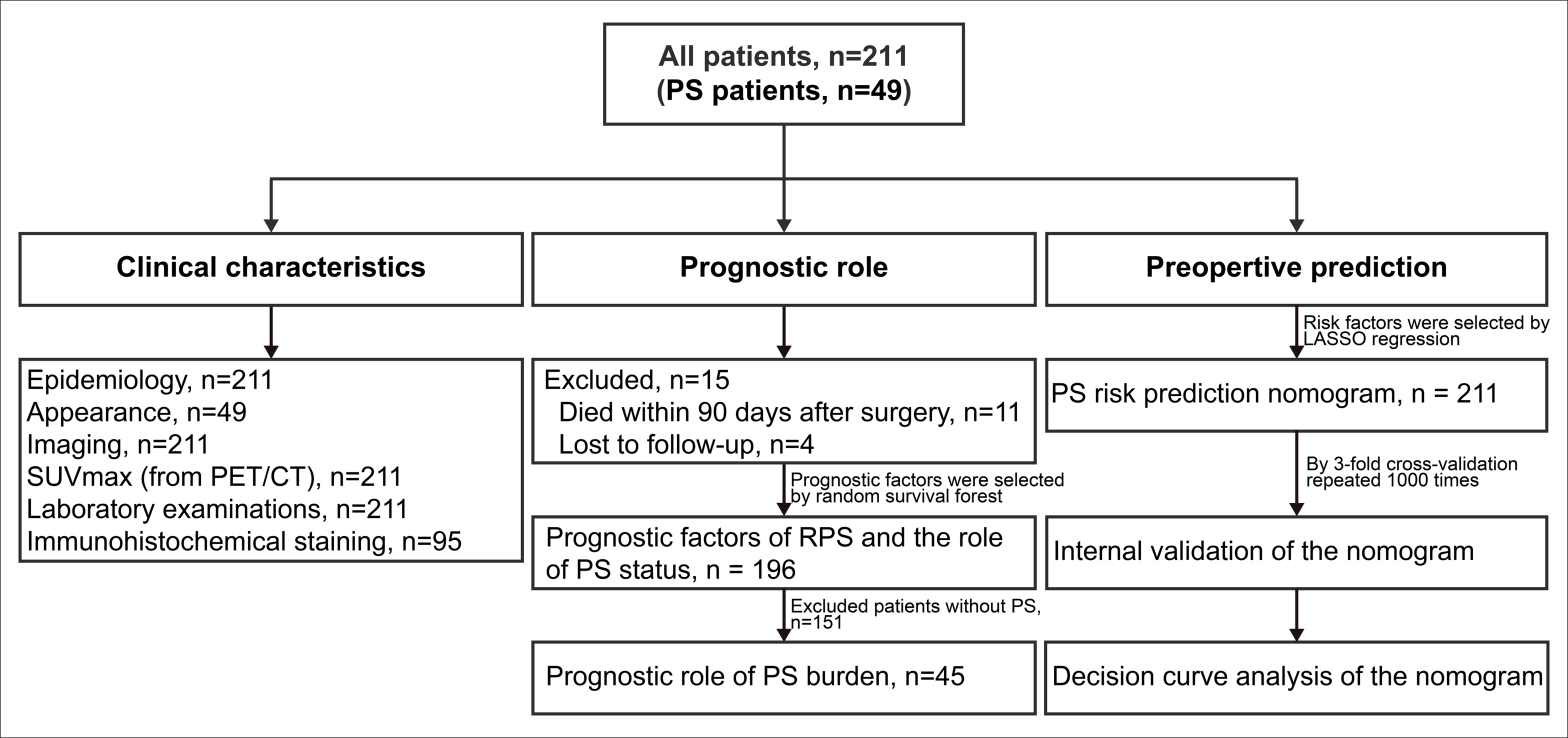
Flowchart of the study design. PS, peritoneal sarcomatosis; RPS, retroperitoneal sarcoma; PET/CT, positron emission tomography/computed tomography; SUVmax, maximum standardized uptake value.

We retrospectively investigated a consecutive cohort with RPS who underwent surgery between January 2011 and January 2019 at Peking University Cancer Hospital Sarcoma Center. Patients with benign retroperitoneal tumors, gastrointestinal stromal tumors, desmoid‐type fibromatosis, pheochromocytomas/paragangliomas, gynecological sarcomas, prostatic sarcomas, or subtypes other than RPS were excluded. Data on age, sex, body mass index (BMI), presentation status, preoperative imaging examinations (within one month before surgery), preoperative positron emission tomography/computed tomography (PET/CT, within three months before surgery), laboratory examinations (within two weeks before surgery), operation records, postoperative pathological results, and postoperative complications were retrieved from electronic medical records. A double-entry and double-check approach was adopted for data management. The detailed study design was shown in the following flowchart (**Figure 1**). Immunohistochemical (IHC) staining was performed on the tissue specimens of well-differentiated liposarcoma (WDLPS) and dedifferentiated liposarcoma (DDLPS). Two pathologists independently evaluated the percentage of positive cells based on the staining intensity in the sections. The immunoreactive score (IRS) was interpreted as negative (IRS 0–1), mild (IRS 2–4), and strongly positive (IRS 5–12). Patients who received preoperative radiotherapy, chemotherapy, or targeted therapy preoperatively and patients with missing tissue specimen were not included in IHC staining.

Patients were categorized into primary (no operation before admission) and recurrent groups (one or more operations before admission) according to their RPS-associated surgical history. PS nodules were confirmed through intraoperative exploration and postoperative pathological results. The number of nodules was classified as more than 7 (multiple nodules) and less than 7 (limited nodules) owing to their survival differences reported by the previous study (3). Peritoneal carcinomatosis index (PCI) was recorded as previous study (14). Pathological diagnoses were reviewed by two experienced pathologists specializing in sarcomas. The pathological subtypes were classified according to the 2020 World Health Organization criteria for bone and soft tissue tumors (2). The 3-tiered grading system of the Federation Nationale des Centres de Lutte Contre le Cancer (FNCLCC) criteria was used for tumor grading (15). All enrolled patients have completed preoperative abdominopelvic contrast-enhanced computed tomography (CT) examinations in our hospital. The tumor size was measured by preoperative imaging according to the maximum tumor diameter (for only one tumor) or the sum of the maximum tumor diameters (for multiple tumors). The status of PS (called “imaging-PS” for differentiation) and ascites were reviewed based on preoperative CT findings by two experienced radiologists. The radiologists were all blinded to the operative findings. If any discrepancies were noted, the experts discussed and resolved the issue until an agreement was reached.

### Surgical outcomes and follow up

All operations were performed by the same experienced surgical team led by CY Hao, and multi-visceral resection was performed when necessary. The treatment algorithm and surgical procedures were described previously (16). Surgical resection was classified as macroscopically complete (R0 or R1) or incomplete (R2) because the anatomy of RPS makes it questionable to use a reliable microscopic assessment of margins. Postoperative complications occurring within 90 days (POD 90) of the surgery were graded according to Clavien–Dindo classification (17).

Baseline examination of patients was performed by outpatient evaluation after one month postoperatively. The evaluation mainly included physical examination, abdominopelvic contrast-enhanced CT or magnetic resonance imaging. Then they were regularly followed by telephone and outpatient evaluation every 3 months for the first 3 years, and every 6 months thereafter (18).

The primary prognostic endpoint was disease-specific survival (DSS), defined as death due to tumor progression. DSS was calculated from the date of surgery to the date of death or the last date of follow-up as the patients were alive. The secondary prognostic endpoint was recurrence-free survival (RFS), calculated from the date of surgery to the date of diagnosis of locally recurrent/metastatic disease or death whichever was observed first.

### Statistical analysis

The statistical analyses consisted of three parts. The first part was about the statistical description and comparison of the clinicopathological characteristics. Continuous data were described as median (interquartile range [IQR]) and their comparisons were performed with student *t*-test or Wilcoxon rank-sum test, as appropriate. Categorical data were presented as number (percentage) and their comparisons were evaluated using *χ*2 test or Fisher’s exact test, as appropriate. Wilcox rank sum test was used for the comparison of ordered categorical variables (e.g., pathological grade, immunohistochemical staining intensity). To make the full use of the available data, an advanced multiple imputation strategy of missing values was applied (19).

In the second part, the random survival forest (RSF) algorithm and Kaplan-Meier method were used to evaluate the roles of PS status in postoperative DSS and RFS. The accuracy of the RSF model was evaluated based on out-of-bag (OOB) error rates. The prognostic importance of covariables were ordered by their variable importance (VIMP) (20). Significantly prognostic covariables were selected based on the combination of minimal depth and VIMP of the RSF model. Kaplan-Meier analysis and log-rank test were used to compare the survival differences. Optimal survival cut-point for PCI was determined using the maximally selected rank statistics (21).

In the third part, the preoperatively available variables with P < 0.1 in the differential comparisons were included in the least absolute shrinkage and selection operator (LASSO) model based on the one standard error rule with 3-fold cross-validation to reduce feature dimensionality (22, 23). Subsequently, the selected risk factors and the imaging-PS status were combined and used to develop a nomogram based on the whole cohort. The discriminative ability of the nomogram was evaluated by the concordance statistics (C-statistics). A calibration curve was plotted to compare the nomogram-predicted probabilities with the observed outcomes by bootstrapping 2000 resamples, accompanied by the Hosmer-Lemeshow test (24, 25). Then the probability and total points for each patient based on the nomogram was calculated, respectively. The receiver operating characteristic (ROC) curves and Youden index method were used to identify the optimal cut-off point for the nomogram. Patients were stratified into high-risk and low-risk groups based on the cut-off point. The improvement on diagnostic values of the nomogram compared with the strategy of imaging-PS alone was evaluated by the area under curves (AUC, equal to C-statistics) of ROC curves and their significant level (P value). The clinical practicability of the nomogram was evaluated by decision curves analysis (DCA). The comparison of clinical practicability between the nomogram and the strategy of imaging-PS alone was evaluated by their net benefits of risk thresholds and the AUC of DCA curves (26). To verify the reliability of the nomogram, we evaluated the changes of C-statistics and R2 by 3-fold repeated 1000 times cross-validation. Each calculation could generate a pair of C-statistics and R2 values (3 × 1000 pairs). Then the mean of C-statistics and R2 was calculated to compare with the original nomogram model (27). The lower the differences between the validation results and the original results, the more reliable the nomogram.

All statistical analyses were performed using R version 4.0.5 (http://www.r-project.org/) with packages of “mice”, “survival”, “survminer”, “randomForest”, “glmnet”, “rms”, “pROC”, “ggDCA”, and “DynNom”. Statistical significance was set at a two-sided P < 0.05.

## Results

### Clinicopathological characteristics

**Figure 2:**
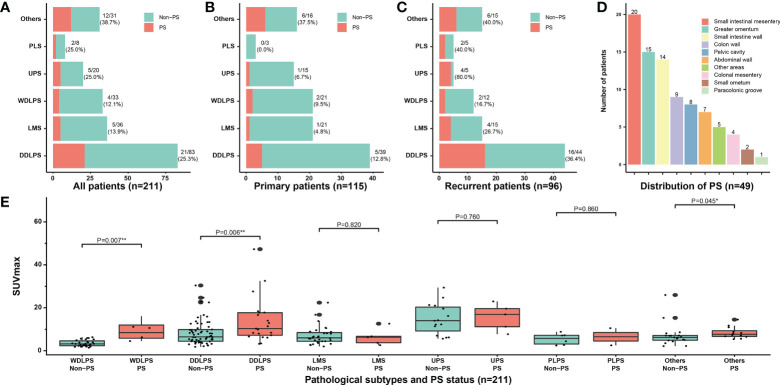
Clinicopathological characteristics of PS. Correlation between the incidence of PS and different pathological subtypes in **(A)** all patients, **(B)** primary patients, and **(C)** recurrent patients; **(D)** The distribution of PS nodules observed by surgical exploration; **(E)** Subgroup analysis of SUVmax according to pathological subtypes and PS status. PS, peritoneal sarcomatosis; WDLPS, well-differentiated liposarcoma; DDLPS, dedifferentiated liposarcoma; LMS, leiomyosarcoma; UPS, undifferentiated pleomorphic sarcoma; PLS, pleomorphic liposarcoma; SUVmax, maximum standardized uptake value. * P<0.05, ** P<0.01.

The clinicopathological characteristics of all patients are presented in **Table 1**. A total of 211 patients were included in this study (108 men and 103 women; median age, 55 years; range, 16–86 years). A total of 18 pathological subtypes of RPS were identified, and the five most common types were WDLPS, DDLPS, leiomyosarcoma, undifferentiated pleomorphic sarcoma (UPS), and pleomorphic liposarcoma. Other relatively uncommon pathological subtypes were shown in **Supplementary Table S1**. Among the 211 patients, 115 (54.5) and 96 (45.5%) patients were categorized into the primary and recurrent groups, respectively. PS was confirmed in 49 (49/211, 23.2%) patients by operative findings and pathological results, and the corresponding incidence was 13.0% (15/115) in the primary group and 35.4% (34/96) in the recurrent group, respectively (P < 0.001). The incidence of PS was higher in the patients with DDLPS and UPS than in those with the other common subtypes. In all common subtypes, the incidence of PS increased with tumor recurrence. This was most significant in patients with UPS, from 6.7% (1/15) to 80% (4/5) (**Figures 2A–C**). The FNCLCC grade is higher in patients with PS compared with patients without PS (P < 0.001). Additionally, 79.6% (39/49) of patients with PS had limited distribution of nodules (≤7), while in 20.4% (10/49) patients more than 7 nodules were detected during surgery. The median PCI of patients with PS was 8 (IQR, 4–14). The nodules of PS most appeared on the surface of the small intestinal mesentery, followed by regions such as the greater omentum, small intestine wall, colon wall, pelvic cavity, and abdominal wall, etc. (**Figure 2D**). The maximum standardized uptake value (SUVmax) of preoperative PET/CT from patients with RPS was analyzed. The median SUVmax was 8.4 (IQR, 6.5–14.0) in the patients with PS, while it was 5.9 (IQR, 3.8–8.9) in the patients without PS (P < 0.001). Subgroup analysis indicated that in most pathological subtypes, the preoperative SUVmax of patients with PS showed an upward trend, especially in WDLPS and DDLPS (P < 0.05, **Figure 2E**). In addition, the main preoperative laboratory examinations of patients were analyzed. The results showed that the peripheral blood platelet and fibrinogen in patients with PS increased significantly, while hemoglobin and albumin decreased significantly (P < 0.05). The operative data of the patients and short-term outcomes are presented in **Table 2**. The most common resected organs included colon/rectum, kidney, pancreas and major vessels. The R0/R1 resection rate was 69.4% and 95.7% (P < 0.001) in PS and non-PS patients, respectively. The operative time, median number of resected organs, and postoperative major complications were comparable between two cohorts.

**Table 1:** Clinicopathological characteristics of the patients.

Parameter	Total Patients(n=211)	PS Patients(n=49)	Non-PS Patients(n=162)	P
Age (years)	55 [46–63]	50 [43–60]	57 [47–64]	**0.022**
Sex				0.201
Male	108 (51.2%)	29 (59.2%)	79 (48.8%)	
Female	103 (48.8%)	20 (40.8%)	83 (51.2%)	
BMI (kg/m^2^)	22.6 [20.8–25.9]	22.6 [20.7–26.2]	22.6 [20.8–25.8]	0.767
Pathological subtypes				0.133
DDLPS	83 (39.3%)	21 (42.9%)	62 (38.3%)	
WDLPS	33 (15.6%)	4 (8.2%)	29 (17.9%)	
LMS	36 (17.1%)	5 (10.2%)	31 (19.1%)	
UPS	20 (9.5%)	5 (10.2%)	15 (9.3%)	
PLS	8 (3.8%)	2 (4.1%)	6 (3.7%)	
Others	31 (14.7%)	12 (24.5%)	19 (11.7%)	
FNCLCC grade				**<0.001**
G1	32 (15.2%)	3 (6.1%)	29 (17.9%)	
G2	90 (42.7%)	15 (30.6%)	75 (46.3%)	
G3	89 (42.3%)	31 (63.3%)	58 (35.8%)	
Tumor size (cm)	20.0 [13.0–29.2]	27.0 [17.0–36.0]	18.0 [12.0–26.0]	**<0.001**
Presentation status				**<0.001**
Primary	115 (54.5%)	15 (30.6%)	100 (61.7%)	
Recurrent	96 (45.5%)	34 (69.4%)	62 (38.3%)	
Previous surgery times				**<0.001**
0	115 (54.5%)	15 (30.6%)	100 (61.7%)	
1	61 (28.9%)	20 (40.8%)	41 (25.3%)	
2	19 (9.0%)	9 (18.4%)	10 (6.1%)	
≥3	16 (7.6%)	5 (10.2%)	11 (6.8%)	
Ascites				**<0.001**
Yes	32 (15.2%)	19 (38.8%)	13 (8.0%)	
No	179 (84.8%)	30 (61.2%)	149 (92.0%)	
SUVmax	6.4 [4.3–10.5]	8.4 [6.5–14.0]	5.9 [3.8–8.9]	**<0.001**
Number of nodules				
>7		10 (20.4%)		
≤7		39 (79.6%)		
PCI		8 [4–14]		
WBC (10 × 10^9^/L)	6.2 [4.8–7.7]	6.1 [4.7–8]	6.2 [4.9–7.6]	0.564
Neutrophils (10 × 10^9^/L)	3.9 [3–5.4]	3.9 [3–5.6]	4 [3–5.3]	0.541
Lymphocyte (10 × 10^9^/L)	1.4 [1–1.7]	1.2 [1–1.6]	1.4 [1.1–1.7]	0.192
Platelet (10 × 10^9^/L)	250 [199–322.5]	299 [215–366]	244 [196.2–304.8]	**0.014**
PWR	40.7 [32–52.8]	41.8 [30.4–59.2]	40.2 [32.3–51.8]	0.392
NWR	0.7 [0.6–0.7]	0.7 [0.6–0.8]	0.7 [0.6–0.7]	0.153
NLR	2.7 [2–4.7]	3.3 [2.2–6.7]	2.7 [2–4.3]	0.149
Hemoglobin (g/L)	121 [106–132.5]	112 [94–123]	122.5 [109.5–133]	**0.001**
Albumin/Globulin	1.6 [1.3–1.9]	1.6 [1.2–1.9]	1.6 [1.4–1.9]	0.232
Albumin (g/L)	39.3 [35–42]	35 [33–40.1]	40 [36.6–42.6]	**0.001**
Fibrinogen (g/L)	4.4 [3.1–6]	5.2 [3.6–6.9]	4.2 [3–5.9]	**0.048**

PS, peritoneal sarcomatosis; BMI, body mass index; WDLPS, well-differentiated liposarcoma; DDLPS, dedifferentiated liposarcoma; LMS, leiomyosarcoma; UPS, undifferentiated pleomorphic sarcoma; PLS, pleomorphic liposarcoma; FNCLCC, Federation Nationale des Centres de Lutte Contre le Cancer; SUVmax, maximum standardized uptake value; PCI, peritoneal carcinomatosis index; WBC, white blood cell; PWR, platelet to white blood cell ratio; NWR, neutrophils to white blood cell ratio; NLR, neutrophil to lymphocyte ratio.

Bold values means P values <0.05.

**Table 2:** Operative data and short-term outcomes.

Parameter	Total Patients(n=211)	PS Patients(n=49)	Non-PS Patients(n=162)	P
Operative time (min)	430 [328–539]	440 [330–600]	425 [326–520]	0.256
Esimated blood loss (ml)	1000 [400–2500]	1100 [500–2800]	1000 [400–2100]	0.233
Multi-visceral resection				0.479
Yes	206 (97.6%)	49 (100%)	157 (96.9%)	
No	5 (2.4%)	0 (0%)	5 (3.1%)	
Completeness of surgery				**<0.001**
Complete resection (R0/R1)	189 (89.6%)	34 (69.4%)	155 (95.7%)	
Incomplete resection (R2)	22 (10.4%)	15 (30.6%)	7 (4.3%)	
Number of resected organs	5.0 [3.0–8.0]	4.0 [3.0–7.0]	5.5 [3.0–8.0]	0.118
Colon/rectum	134 (63.5%)	32 (65.3%)	102 (63.0%)	
Kidney	100 (47.4%)	14 (28.6%)	86 (53.1%)	
Pancreas	65 (30.8%)	11 (22.4%)	54 (33.3%)	
Small intestine	40 (19.0%)	17 (34.7%)	23 (14.2%)	
Liver	19 (9.0%)	5 (10.2%)	14 (8.6%)	
Spleen	44 (20.9%)	11 (22.4%)	33 (20.4%)	
Stomach	41 (19.4%)	7 (14.3%)	34 (21.0%)	
Major vessels	45 (21.3%)	7 (14.3%)	38 (23.5%)	
POD 90 complications grade				0.476
No complication	118 (55.9%)	24 (49.0%)	94 (58.0%)	
Clavien-Dindo I–II	41 (19.4%)	12 (24.5%)	29 (17.9%)	
Clavien-Dindo III–V	52 (24.6%)	13 (26.5%)	39 (24.1%)	

PS, peritoneal sarcomatosis; POD, postoperative day.

Bold values means P values <0.05.

**Figure 3:**
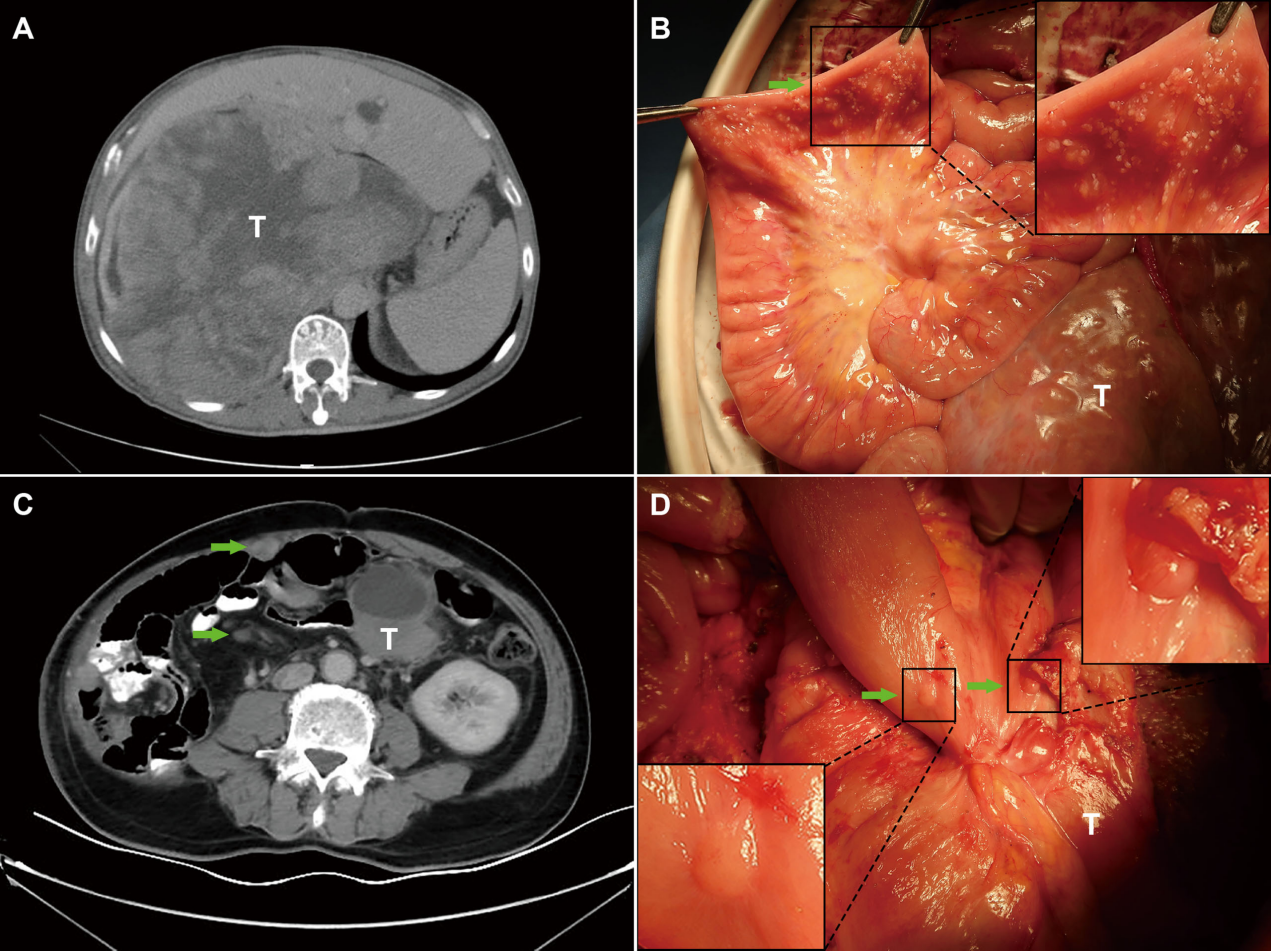
Representative patients with undetectable and detectable PS by preoperative imaging. **(A)** The PS status of patient 1 was failed to be detected by preoperative imaging; **(B)** PS was incidentally detected during the surgery; **(C)** The PS status of patient 2 was successfully detected by preoperative imaging; **(D)** PS was confirmed during the surgery. T, primary tumors; Green arrows indicate PS nodules; Boxed regions are shown as magnified images in the inset.

From 211 patients with RPS, 47 patients were diagnosed with PS by preoperative CT (imaging-PS), of which 35 patients were confirmed by pathology. The positive predictive value was 74.5% (35/47); the negative predictive value was 91.5% (150/164); the diagnostic sensitivity was 71.4% (35/49); the specificity was 92.6% (150/162). Among 115 patients in the primary group, 14 patients were diagnosed as imaging-PS, of which 10 patients were confirmed by pathology. The positive predictive value was 71.4% (10/14); the negative predictive value was 95.0% (96/101); the diagnostic sensitivity was 66.7% (10/15); the specificity was 96.0% (96/100). Among the 96 patients in the recurrent group, 33 patients were diagnosed as imaging-PS, of which 25 patients were confirmed by pathology. The positive predictive value was 75.8% (25/33); the negative predictive value was 85.7% (54/63); the diagnostic sensitivity was 73.5% (25/34); the specificity was 87.1% (54/62) (**Table 3**). Representative patients with detectable and undetectable PS by preoperative CT alone were listed in **Figure 3**, respectively.

**Table 3:** Preoperative peritoneal sarcomatosis identified by computed tomography (CT).

Cohort groups		Actual PS (+)	Actual PS (-)	Total
All patients (n=211)	Imaging-PS (+)	35	12	47
	Imaging-PS (-)	14	150	164
	Total	49	162	211
Primary group (n=115)	Imaging-PS (+)	10	4	14
	Imaging-PS (-)	5	96	101
	Total	15	100	115
Recurrent group (n=96)	Imaging-PS (+)	25	8	33
	Imaging-PS (-)	9	54	63
	Total	34	62	96

PS, peritoneal sarcomatosis.

**Figure 4:**
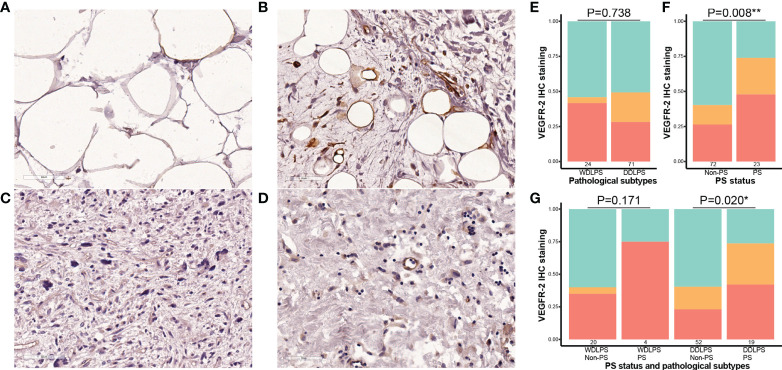
Correlation between the expression of VEGFR-2 and pathological subtypes and PS status in retroperitoneal liposarcoma. All microscopic images were acquired with 200 × magnification. **(A)** Cases with negative expression of VEGFR-2 in WDLPS; **(B)** Cases with positive expression of VEGFR-2 in WDLPS; **(C)** Cases with negative expression of VEGFR-2 in DDLPS; **(D)** Cases with positive expression of VEGFR-2 in DDLPS; **(E)** The staining intensity of VEGFR-2 in WDLPS and DDLPS (red is strongly positive, orange is weakly positive, and green is negative.); **(F)** the staining intensity of VEGFR-2 in cases without PS and in cases with PS; **(G)** the staining intensity of VEGFR-2 in different liposarcoma subtypes and PS status. PS, peritoneal sarcomatosis; WDLPS, well-differentiated liposarcoma; DDLPS, dedifferentiated liposarcoma. * P<0.05, ** P<0.01.

Finally, IHC staining was performed in paraffin sections from 95 eligible patients. The clinicopathological characteristics of patients are shown in **Supplementary Table S2**. The results showed that the positive expression rate of VEGFR-2 was 45.8% (11/24) in WDLPS and 49.3% (35/71) in DDLPS. There was no significant difference between the two pathological subtypes (P = 0.736). The positive expression rate of VEGFR-2 was 73.9% (17/23) in patients with PS and 40.3% (29/72) in patients without PS. The expression of VEGFR-2 in the PS group was significantly higher than that in the non-PS group (P = 0.008). Further subgroup analysis showed that no matter in patients with WDLPS or DDLPS, the expression of VEGFR-2 in patients with PS was higher. This trend was more significant in patients with DDLPS (P = 0.020) (**Figure 4**).

### Prognostic role

**Figure 5:**
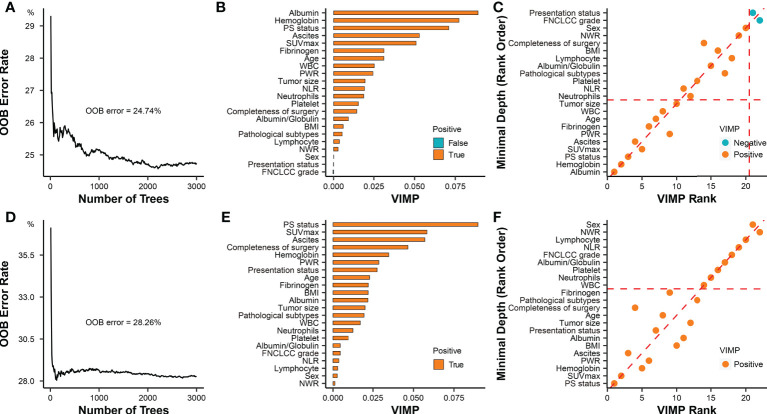
Prognostic role of PS status in RPS. **(A)** Error rates of the random survival forest model for evaluating DSS including all covariables. **(B)** The importance of all covariables based on the random survival forest model for evaluating DSS. The covariates filled with orange (“True”) have a positive effect on the model, while the covariates filled with green (“False”) have a negative effect on the model. The importance of covariates is elevated with the increase of VIMP score. **(C)** The selection of covariables was based on combined minimal depth and VIMP approaches of random survival forest model for evaluating DSS. Covariables in the rectangular box consisting of coordinate axes and dashed auxiliary lines (red) were selected as significant prognostic factors. **(D)** Error rates of the random survival forest model for evaluating RFS including all covariables; **(E)** The importance of all covariables was ordered by the random survival forest model for evaluating RFS; **(F)** The selection of covariables was based on combined minimal depth and VIMP approaches of random survival forest model for evaluating RFS. Covariables under the horizontal dashed auxiliary line (red) were selected as significant prognostic factors. OOB, out-of-bag; VIMP, variable importance; PS, peritoneal sarcomatosis; SUVmax, maximum standardized uptake value; NLR, neutrophil to lymphocyte ratio; PWR, platelet to white blood cell ratio; WBC, white blood cell; NWR, neutrophils to white blood cell ratio; BMI, body mass index; FNCLCC, Federation Nationale des Centres de Lutte Contre le Cancer.

Among the 211 patients, 196 (92.9%) were included in the survival analysis, and 15 patients were excluded because of loss to follow-up (n=4; none patients had PS) or death within 90 days postoperatively (n =11; 4 patients had PS). The OOB error of the RSF model was 24.74% and 28.26% for DSS and RFS in the whole cohort, respectively (**Figures 5A, D**). Based on the RSF algorithm, the prognostic importance of these covariables was ordered, and PS status ranked the third in DSS, and played the most important role in RFS (**Figures 5B, E**). The consistent covariables vital to the DSS included albumin, hemoglobin, PS status, SUVmax, ascites, platelet to white blood cell ratio (PWR), fibrinogen, age, white blood cell, and tumor size (**Figure 5C**). The consistent covariables vital to the RFS included PS status, SUVmax, hemoglobin, PWR, ascites, BMI, albumin, presentation status, tumor size, age, completeness of surgery, pathological subtypes, and fibrinogen (**Figure 5F**).

**Figure 6:**
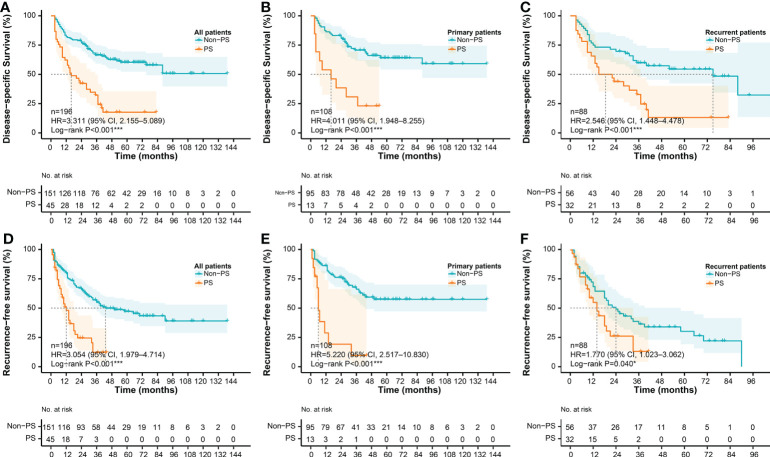
Survival differences according to PS status. DSS according to PS status in **(A)** the whole cohort, **(B)** the primary cohort, and **(C)** the recurrent cohort, respectively; RFS according to PS status in **(D)** the whole cohort, **(E)** the primary cohort, and **(F)** the recurrent cohort, respectively. DSS, disease-specific survival; RFS, recurrence-free survival; PS, peritoneal sarcomatosis.

The median DSS in the whole cohort (n = 196) was 75.0 months (95% CI, 37.0–NA), and the estimated 3- and 5-year DSS rates were 58.6% and 50.3%, respectively. The median RFS in the whole cohort was 33.0 months (95% CI, 25.0–50.0), and the estimated 3- and 5-year RFS rates were 46.4% and 39.9%, respectively. Survival analysis showed a significantly worse DSS in the patients with PS (n = 45) than that in the patients without PS (n = 151), regardless of the presentation status (log-rank P < 0.001). The median DSS of the PS patients was 16.0 months (95% CI, 11.0–35.0), and the estimated 3- and 5-year DSS rates were 32.3% and 0%, respectively. In contrast, the median DSS of the patients without PS was not reached (95% CI, 75.0–NA), and the estimated 3- and 5-year DSS rates were 66.5% and 60.3%, respectively (**Figures 6A–C**). Similarly, patients with PS are more inclined to have postoperative recurrence than patients without PS, especially in the primary group (log-rank P < 0.001). The median RFS of the patients with PS was 12.0 months (95% CI, 8.0–18.0), and the estimated 3- and 5-year RFS rates were 11.2% and 0%, respectively. In contrast, the median RFS of the patients without PS was 43.0 months (95% CI, 33.0–NA), and the estimated 3- and 5-year RFS rates were 55.0% and 47.2%, respectively. (**Figures 6D–F**)

Patients with PS nodules > 7 had slightly poor survival compared with those with ≤ 7 nodules (median DSS, 10.0 vs. 23.0 months), but the statistical difference was not significant (log-rank P = 0.117) (**Supplementary Figure S1A**). Other thresholds of nodules (from 3 to 20) were also evaluated, but no significant result was produced. The optimal survival cut-off point for PCI was determined to be 16, and significant difference was observed in survival analysis (log-rank P = 0.001). The DSS of patients with PCI more than 16 was significantly worse than that with PCI less than 16 (median DSS, 6.5 vs. 23.0 months). The 1-year DSS rates were 16.7% (>16) and 69.2% (≤16), respectively (**Supplementary Figure S1B**).

### Preoperative prediction

**Figure 7:**
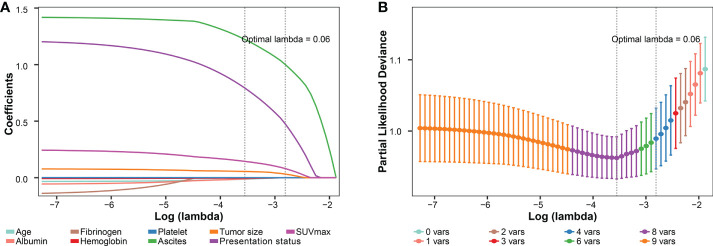
Preoperative variables selected using the LASSO regression and cross-validation. **(A)** The coefficients of covariables in LASSO regression model. **(B)** Tuning parameter (lambda) selection using 3-fold cross-validation. LASSO, the least absolute shrinkage and selection operator; SUVmax, maximum standardized uptake value.

**Figure 8:**
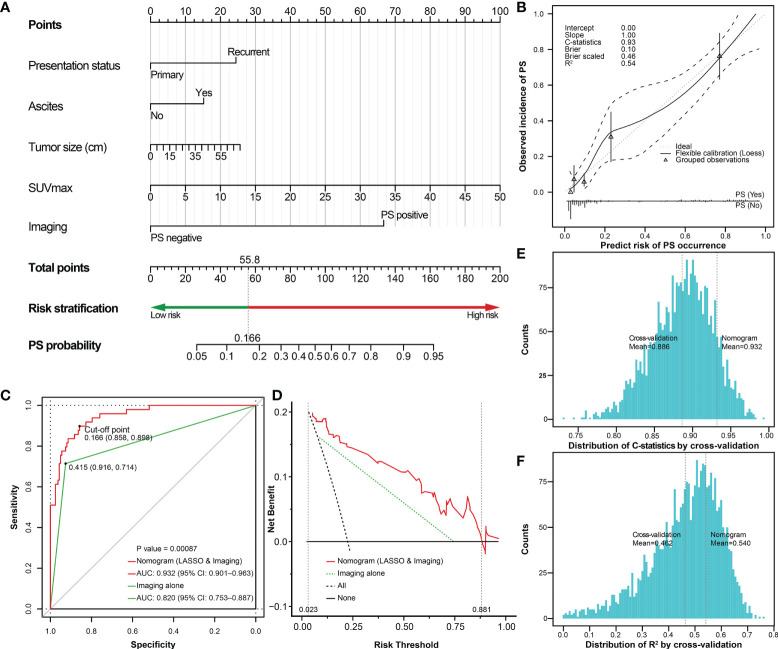
The nomogram predicting the probability of PS based on selected risk factors and preoperative imaging. **(A)** The nomogram estimating the risk of PS established for the whole cohort; **(B)** The calibration plot of the nomogram; **(C)** The ROC curves shows that performance of the nomogram is superior to imaging alone. The risk of PS could be classified into low-risk (<0.166) and high-risk (≥0.166) stratifications. **(D)** DCA plot shows the clinical net benefit of different prediction models. The risk thresholds between the two dashed auxiliary lines (grey) are the most applicable range of the nomogram. The histogram shows the distribution of **(E)** C-statistics and **(F)** R^2^ after internal cross-validation based on 3-fold repeated 1000 times, respectively. The two auxiliary lines (grey) refer to the C-statistics or R^2^ of the internal cross-validation and the original nomogram, respectively. PS, peritoneal sarcomatosis; SUVmax, maximum standardized uptake value; LASSO, the least absolute shrinkage and selection operator; ROC, receiver operating characteristic; DCA, decision curves analysis; CI, confidence interval; C-statistics, concordance statistics.

To improve the prediction rate of PS by preoperative imaging alone, we explored other potential risk factors based on the LASSO regression and 3-fold cross-validation. We got the four most significant risk factors: ascites, presentation status (primary vs. recurrent), SUVmax, and tumor size (**Figure 7**). For predicting PS probability, a nomogram including the above four risk factors and imaging-PS was developed based on the whole cohort (n = 211) (**Figure 8A**). The C-statistics of the nomogram in discriminating PS was 0.932 (95% confidence interval [CI], 0.901–0.963) and 0.820 (95% CI, 0.753–0.887) for the imaging-PS alone. After 2000 bootstrapping resamples, the solid curve of the calibration plot is very close to the ideal line (dotted line), suggesting that the predicted probabilities and the observed outcomes are in good agreement (**Figure 8B**). The Hosmer-Lemeshow test yielded a P-value of 0.317, indicating that the nomogram fitted well. The AUC values (equal to the C-statistics) of the ROC curves showed that the nomogram was significantly superior to the imaging-PS alone (AUC: 0.932 vs. 0.820, **Figure 8C**, P < 0.001). The optimal thresholds of the probability and total points in the nomogram were identified as 0.166 and 55.8, respectively. These two thresholds were corresponding to each other and were used to classify patients into “low risk” and “high risk” strata.

Subsequently, the DCA was performed to analyze the clinical practicability of the nomogram. For our model, when the predicted probability threshold was set from 0.023 to 0.881, the clinical net benefits were positive. The AUC of the DCA in our nomogram was 0.095, while it was 0.053 according to the imaging-PS status alone. The results showed that our nomogram had a superior net benefit to the imaging-PS status by CT alone (**Figure 8D**). To verify the reliability of the nomogram, 3-fold repeated 1000 times cross-validation was performed. The mean of C-statistics and R^2^ of the cross-validation results was 0.886 (95% CI, 0.885–0.888) and 0.462 (95% CI, 0.457–0.467), respectively. Compared with the original nomogram, the validation results were on the brink of that of the original nomogram, which suggests the nomogram is reliable (**Figures 8E, F**). To further simplify the calculation, the nomogram was developed into an online nomogram (https://sarcoma52.shinyapps.io/PSprediction/) (**Supplementary Figure S2**).

**Figure 9:**
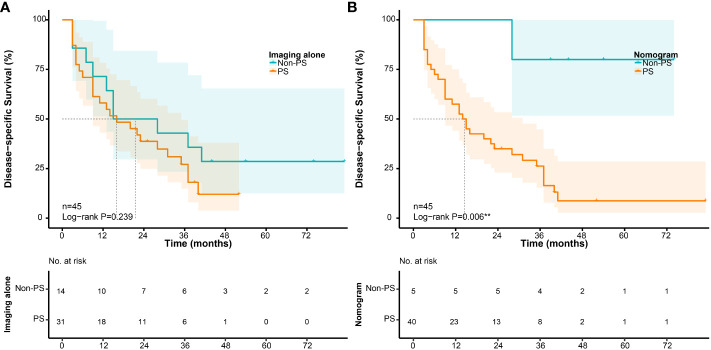
Survival differences in patients with PS according to their preoperative evaluation. **(A)** Disease-specific survival according to preoperative imaging alone; **(B)** Disease-specific survival according to the nomogram.

According to the cut-off point, the sensitivity and specificity of the nomogram to detect PS were 89.8% (44/49) and 85.8% (139/162), and the positive and negtive predictive value were 65.7% (44/67) and 96.5% (139/144), respectively. Compared to preoperative imaging alone, the nomogram significantly improved the diagnostic sensitivity from 71.4% (35/49) to 89.8% (44/49). In addition, to further explore the potential clinical value of the proposed nomogram, we compared the survival differences between unexpected PS and PS identified preoperatively in depth. Using preoperative imaging alone, the prognosis of patients with PS (+) (n=31) was slightly worse than that of patients with PS (-) (n=14)(P=0.239, **Figure 9A**). However, when using the proposed nomogram, the prognosis of patients classified as high-risk of PS (n=40) was significantly worse than that of patients with low-risk of PS (n=5)(P=0.006, **Figure 9B**).

## Discussion

RPS is an insidious disease with a large volume and various histological subtypes. Theoretically, RPS originates from and should be restricted to the retroperitoneal space. However, sometimes the anatomical boundary is broken through spontaneously or iatrogenically, which causes PS, presenting as lesions occurring on the peritoneal surface or intraperitoneal viscera (10). As peritoneal surface malignancy, current knowledge of the management of PS was mainly from peritoneal carcinomatosis (PC), a more common disease caused by gastrointestinal carcinoma. However, the differences in biological behavior between RPS and carcinoma should not be ignored. So far, research on the clinicopathological characteristics, the prognostic role, and the preoperative evaluation of PS has been insufficient. Therefore, it is necessary to conduct this research and attempt to clarify these issues.

Although PS may be an accidental phenomenon in the initial diagnosis, actually its incidence is not very rare during the development process of RPS. In the current cohort, the incidence of PS was 13.0% (15/115) in the primary group and 35.4% (34/96) in the recurrent group, which is similar to the literature (6, 7). PS nodules most appear on the small intestinal mesentery, followed by the greater omentum and small intestine wall. The most common sites were the covered peritoneum and the areas related to organ mobility, where exfoliated tumor cells are more likely to adhere. Besides, unlike the diffusely miliary distribution of PC, the distribution of PS is often nodular and limited (79.6%), making it more possible to remove all visible tumors by surgery. In addition, we found that the occurrence of PS is closely associated with the degree of malignancy of the primary tumors. The FNCLCC grade and SUVmax tend to be higher in patients with PS than that in patients without PS. PS is more likely to occur in DDLPS and UPS, which are typically subtypes with higher malignancy. In addition, the peripheral blood platelet and fibrinogen increased significantly in patients with PS, while hemoglobin and albumin decreased significantly in patients with PS. Previous studies found that the peripheral blood platelet and fibrinogen are significantly related to the progress of malignant tumors (28–30), while the development of malignancies is related to the decrease in the hemoglobin and albumin (31, 32). Thus, these changes may contribute to the progress of RPS as well.

On the other hand, the present study focused on patients with WDLPS and DDLPS to analyze the correlation between the expression of VEGFR-2 and PS status by IHC staining. The results suggested that the expression of VEGFR-2 in the primary tumors of WDLPS and DDLPS with PS was higher than that in tumors without PS, especially in DDLPS (P = 0.020). In the VEGFR family, VEGFR-2 is considered as the most critical factor to promote angiogenesis (33). Studies have shown that tyrosine kinase inhibitors targeting VEGFR-2 could inhibit tumor angiogenesis and tumor growth (34, 35), and could also directly induce autophagy and apoptosis of tumor cells (36, 37). Therefore, these findings may indicate that patients with PS may benefit more significantly from anti-VEGFR-2 targeted therapies.

PS often indicates advanced disease with poor survival. Some studies revealed that the median overall survival (OS) of patients with PS was between 6.0−14.0 months (38, 39). Similarly, in the present cohort, no matter in the primary or recurrent group, the median DSS of patients with PS was significantly worse than that of patients without PS (**Figures 6A–C**, P <0.001). According to the two RSF models (DSS and RFS) in the current study, PS status was one of the most important prognostic factors in patients with RPS. The burden of PS was also regarded as an important prognostic parameter in patients with PS, such as the number of nodules and PCI (3, 14). Anaya et al. found that patients with more than 7 tumors have the worst prognosis (3). In the current study, we did not observe a significant survival difference in the number of nodules. However, we found that the optimal survival cut-off point of PCI is 16, which could stratify patients with significant statistical difference.

With the consideration of the significant prognostic role as well as unsatisfactory preoperative detection rate of PS, we then focused on exploring other risk factors to improve the prediction rate of preoperative imaging alone. According to previous literature, the preoperative detection rate of PS was considered unsatisfactory, especially for minor nodules (11). In the current study, only 71.4% (35/49) of patients with PS can be detected by CT scan preoperatively. This could be due to the presence of small nodules that were difficult to detect, and as a result of the abdominal anatomy itself. An RPS with large tumor size could lead to the squeezing and twisting of the abdomen, and the nodules could be obscured in imaging. In addition, if the patient underwent previous surgery, it was more likely to change the peritoneal structure and form adhesion, cords, and other non-tumor structures. Therefore, other predictors need to be explored to improve the detection rate. In the present study, the LASSO regression based on cross-validation was utilized to select the preoperatively available risk factors of PS. As a result, four risk factors including presentation status (primary vs. recurrent), ascites, SUVmax, and tumor size were selected. Ascites is a common sign when the peritoneum is involved, and recurrent disease is considered to have a obviously poorer prognosis in RPS (40). SUVmax is reported to may predict the proliferative potential of soft tissue sarcomas (41), and tumor size is commonly known as a key prognostic factor to RPS. We combined them with imaging-PS and developed a visualized nomogram. The nomogram showed a good and reliable prediction ability, which increased the diagnostic sensitivity of PS from 71.4% (35/49) to 89.8% (44/49) compared to preoperative imaging alone. It means that 9 out of 14 patients with PS who were not detected by preoperative imaging were identified as PS(+) by the nomogram. For example, as presented in **Figures 3A, B**, the PS status was not found by preoperative CT, but the patient had a total point of 60.6 (corresponding probability = 0.189) according to the nomogram. Therefore, the patient should be classified as a high-risk patient.

The proposed nomogram may improve the patient selection and provide potential clinical value. In the further exploration, the nomogram showed superior stratification ability than preoperative imaging alone in patients with PS. The prognosis of patients classified as high-risk was significantly worse than that of patients with low-risk (P=0.006, **Figure 9B**). It would mean that it may be better to use the proposed nomogram before surgery to predict the effect of surgical treatment for patients with PS. For patients with low-risk of PS according to the nomogram, the effect of surgical treatment may be better even if PS is found accidentally during surgery subsequently. The optimal treatment modality of PS remains controversial. In the past, due to the overall limited effect of chemotherapy on most of subtypes of RPS, surgery was almost the only way. Several previous studies revealed that more than 60% of patients with PS could achieve macroscopically complete resection, and their survival was significantly improved compared with incomplete resection (6, 42, 43). However, the overall limited survival benefit and high morbidity could not be ignored. Moreover, according to the consensus from TARPSWG, surgery for PS should be restricted to palliative intervention according to the symptoms (10). Some new agents have shown therapeutic effects against sarcomas, including Eribulin, novel tyrosine kinase inhibitors, and immune checkpoint inhibitors (44–46). Therefore, preoperative accurate prediction of PS is helpful for comprehensive evaluation and decision-making. The nomogram may contribute to screening patients to adopt more appropriate therapeutic approaches or participate in clinical trials.

The current study has certain limitations. First, the retrospective design may produce selective biases and information missing compared with a prospective design. For example, some socioeconomic/demographic factors with impacts on outcomes were not included in this study (47). Second, due to the low incidence of RPS, the sample size is relatively limited. Further multicenter, large-scale cohorts are required to verify our findings. Third, although the prediction model performed well in the internal cross-validation, further external validation is yet required to confirm its reliability.

## Conclusion

PS is one of the most significant prognostic predictors in patients with RPS, and it occurs more often in recurrent RPS and in RPS with higher malignant tendency. The expression of VEGFR-2 is higher in patients with PS for WDLPS or DDLPS. The proposed nomogram is an effective clinical tool to predict and assess PS preoperatively, which may contribute to clinical decision-making.

## Data availability statement

The raw data supporting the conclusions of this article will be made available by the authors, without undue reservation.

## Author contributions

YL, AL and C-YH contributed to the conception and design of the study. YL, AL and X-YT collected data and performed the statistical analysis. YL wrote the first draft of the manuscript. AL, J-HW, C-PL, B-NL and HQ wrote the sections and reviewed the manuscript. All authors contributed to manuscript revision, read, and approved the submitted version.

## Funding

This study was supported by National Natural Science Foundation of China (approval No. 91959120), Capital Health Research and Development of Special Funds (approval No. 2020-1-1021), Beijing excellent talent training project (approval No.2018000021469G269), China Postdoctoral Science Foundation (approval No. 2020M680260), Beijing Municipal Administration of Hospital’s Ascent Plan (approval No. DFL20181104), and Beijing Municipal Administration of Hospitals’ Youth Program (approval No. QML20181104).

## Acknowledgments

We are very grateful to the patients and investigators who participated in the current study.

## Conflict of interest

The authors declare that the research was conducted in the absence of any commercial or financial relationships that could be construed as a potential conflict of interest.

## Publisher’s note

All claims expressed in this article are solely those of the authors and do not necessarily represent those of their affiliated organizations, or those of the publisher, the editors and the reviewers. Any product that may be evaluated in this article, or claim that may be made by its manufacturer, is not guaranteed or endorsed by the publisher.
