# Supplementary material for: Multidimensional characteristics, prognostic role, and preoperative prediction of peritoneal sarcomatosis in retroperitoneal sarcoma
Source: Front Oncol. 2022 Oct 27;12:950418. doi: 10.3389/fonc.2022.950418 (PMC9646709; doi:10.3389/fonc.2022.950418)
Supplement: Supplementary file 1 [file DataSheet_1.docx]

**Supplementary Material**

**Supplementary Tables**

**Supplementary Table S1 Pathological subtypes of "Others" included in this study.**

| Other Pathological Subtypes | RPS Patients (n=31) | PS Patients (n=12) |
| --- | --- | --- |
| Malignant peripheral nerve sheath tumor | 6 | 1 |
| Synovial sarcoma | 5 | 4 |
| Malignant solitary fibroma | 4 | 1 |
| Inflammatory myofibroblastic tumor | 3 | 0 |
| Rhabdomyosarcoma | 2 | 1 |
| Undifferentiated sarcoma | 2 | 1 |
| Fibrosarcoma | 2 | 0 |
| Ewing sarcoma/primitive neuroectodermal tumor | 2 | 1 |
| Chondrogenic osteosarcoma | 1 | 1 |
| Chondrosarcoma | 1 | 0 |
| Soft tissue angiosarcoma | 1 | 1 |
| Myxofibrosarcoma | 1 | 0 |
| Myxoid/round cell liposarcoma | 1 | 1 |

RPS, retroperitoneal sarcoma; PS, peritoneal sarcomatosis

**Supplementary Table S2 Clinicopathological characteristics of patients who underwent immunohistochemical staining (n=95)**

| Characteristics | n (%) / median [IQR] |
| --- | --- |
| Age (years) | 58 [47–64] |
| Sex |  |
| Male | 49 (51.6) |
| Female | 46 (48.4) |
| Presentation status |  |
| Primary | 48 (50.5) |
| Recurrent | 47 (49.5) |
| Tumor size (cm) | 24 [16–31] |
| FNCLCC grade |  |
| G1 | 20 (21.1) |
| G2 | 47 (49.5) |
| G3 | 28 (29.5) |
| Pathological subtypes |  |
| WDLPS | 24 (25.3) |
| DDLPS | 71 (74.7) |

IQR, interquartile range; FNCLCC, Federation Nationale des Centres de Lutte Contre le Cancer; WDLPS, well-differentiated liposarcoma; DDLPS, dedifferentiated liposarcoma.

**Supplementary Figures**

**
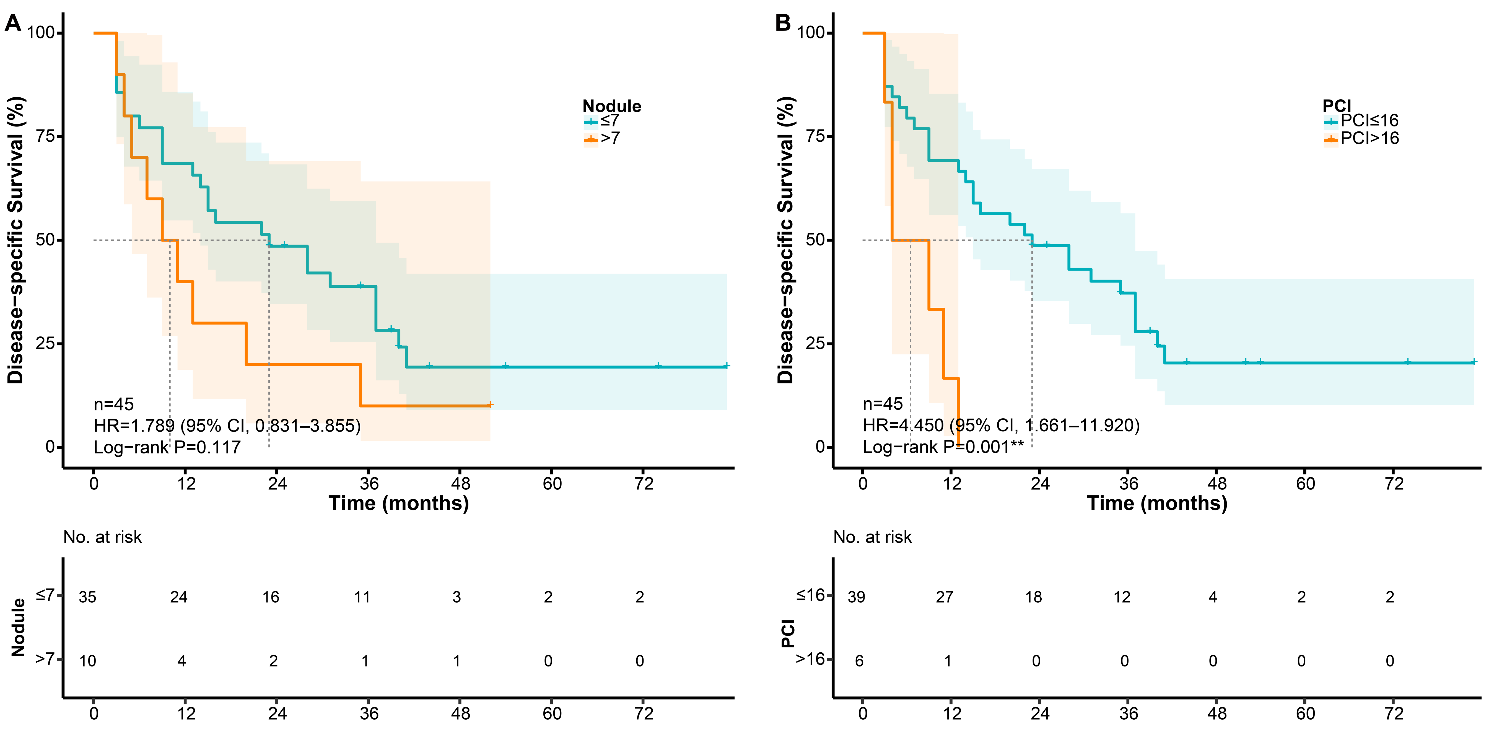
**

**Supplementary Figure S1 Prognostic value of the burden of peritoneal sarcomatosis.** DSS according to **(A)** the number of PS nodules and **(B)** PCI in patients with PS, respectively. DSS, disease-specific survival; PS, peritoneal sarcomatosis; PCI, peritoneal carcinomatosis index.

**
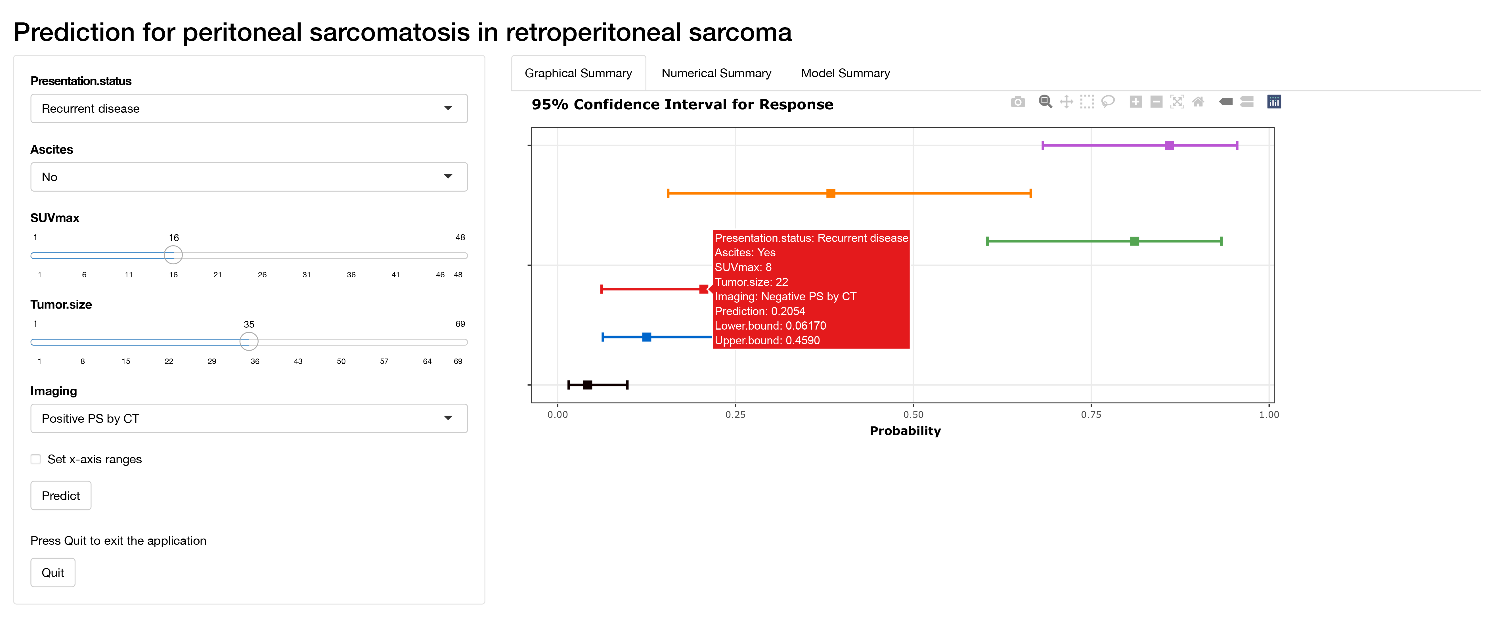
**

**Supplementary Figure S2 Online nomogram.** SUVmax, maximum standardized uptake value; CT, computed tomography.
